# Effects of virtual reality-based pulmonary rehabilitation in patients with chronic obstructive pulmonary disease: A meta-analysis

**DOI:** 10.1097/MD.0000000000036702

**Published:** 2023-12-29

**Authors:** Xiuqin Chai, Lingyun Wu, Zhihong He

**Affiliations:** a Department of Pulmonary and Critical Care Medicine, The Quzhou Affiliated Hospital of Wenzhou Medical University, Quzhou People’s Hospital, Quzhou, Zhejiang, China; b School of Nursing, Zhejiang Chinese Medical University, Hangzhou, Zhejiang, China.

**Keywords:** chronic obstructive pulmonary disease, meta-analysis, pulmonary rehabilitation, virtual reality

## Abstract

**Background:**

Virtual reality (VR)-based pulmonary rehabilitation has been used in the management of chronic obstructive pulmonary disease (COPD). The efficacy of VR-based pulmonary rehabilitation for improving lung function in patients with COPD is controversial. Therefore, the aim of this meta-analysis was to evaluate the efficacy of VR combined with pulmonary rehabilitation for lung function in patients with COPD.

**Methods:**

This study followed the Preferred Reporting Items for Systematic Reviews and Meta-Analyses guidelines. The search was performed in the Cochrane Library, EMBASE, Web of Science, PubMed, and China National Knowledge Infrastructure databases from inception to February 1, 2023. All included studies were randomized controlled trials that assessed VR combined with pulmonary rehabilitation for COPD patients. The effect size was calculated with standardized mean difference (SMD) and its 95% confidence interval (CI). The Cochrane Collaboration tool was used to assess the risk of bias. Publication bias was assessed by Egger test.

**Results:**

A total of 11 studies met the inclusion criteria and were included in this study. The combined effect size showed that VR combined with pulmonary rehabilitation was more effective than pulmonary rehabilitation alone at improving forced expiratory volume in 1 second% (SMD: 0.51; 95% CI 0.19,0.82; *P* = .002), forced expiratory volume in 1 second/forced vital capacity (SMD: 0.71; 95% CI 0.49,0.93; *P* < .001), dyspnea (SMD: −0.44; 95% CI −0.66, −0.22; *P* < .001), and 6-minute walking test (SMD: 059; 95% CI 0.39, 0.79; *P* < .001). In addition, the VR combined with pulmonary rehabilitation improved depression (SMD: −0.34; 95% CI −0.05, −0.03; *P* = .033) and anxiety mood (SMD: −0.57; 95% CI −1.11, −0.04; *P* = .036) compared with the pulmonary rehabilitation group.

**Conclusion:**

This meta-analysis indicated that VR regimens could be used to enhance the therapeutic effect of pulmonary rehabilitation in patients with COPD. However, as a rapidly evolving field, more well-designed randomized controlled trials are needed to determine the impact of VR-based pulmonary rehabilitation on COPD patients.

## 1. Introduction

Chronic obstructive pulmonary disease (COPD) is a progressive respiratory disease characterized by chronic respiratory distress and airflow limitation.^[1,2]^ The World Health Organization estimates that by 2030, COPD will become the third leading cause of death in a word.^[3]^ Chronic and progressive dyspnea is the most typical symptom of COPD, which lead to a decrease in the patient’s mobility, quality of life, and seriously affects the physical and mental health, which imposes a huge burden on society and individuals.^[4,5]^ Pulmonary rehabilitation is considered the common and effective treatment for all COPD patients.^[6]^ Typically, pulmonary rehabilitation program is implemented by a multidisciplinary team, including exercise training, nutritional supplements, and psychological support.^[7]^ Evidence suggests that pulmonary rehabilitation can improve exercise capacity, reduce fatigue, and respiratory distress in COPD patients.^[8,9]^ However, adherence to pulmonary rehabilitation is a critical issue in this population as a result of geographical distance, lack of motivation, inconvenience, and financial struggles.^[10]^ Therefore, searching alternative rehabilitation models may increase the number and scale of beneficiaries.

During the COVID-19 pandemic, the concept of home-based pulmonary rehabilitation has emerged as a promising alternative model to improve uptake and access.^[11]^ Extensive research has demonstrated that home pulmonary rehabilitation is a safe approach that can enhance clinical outcomes even with limited resources.^[12]^ Recognizing the challenges faced by health systems, the World Health Organization recommends the utilization of digital technologies to address these issues effectively.^[13]^ The advent of new digital technologies has opened unique opportunities to implement personalized pulmonary rehabilitation programs at home, catering to the specific needs of each patient.^[14–16]^ The widespread adoption of mobile devices and healthcare platforms has facilitated the use of telerehabilitation approaches, thereby overcoming barriers related to accessibility and cost. This, in turn, has the potential to extend rehabilitation services to a wider population.^[1,17]^ Among the various telerehabilitation approaches, virtual reality (VR) emerges as an innovative solution that enables patients to engage in exercises at home, thereby improving accessibility and promoting a more active lifestyle.^[18,19]^

With these solutions, VR has been explored as a potential adjunct to rehabilitation programs.^[20,21]^ VR is a computer-generated environment that can create a sense of presence using computer electronic information simulation technology.^[22]^ Its immersive interactivity is considered to provide a particularly engaging method, and through its computer-generated interactive environment and experience can increase patients’ subjective initiative through multisensory feedback.^[23,24]^ With the continuous improvement of information technology, VR technology is gradually applied to pulmonary rehabilitation training.^[18,25]^ Limited evidence suggests that VR-based pulmonary rehabilitation can improve lung function, exercise endurance, and dyspnea in COPD patients.^[26–28]^ However, most studies on the effect of VR technology on COPD are mostly small sample sizes, which makes it difficult to draw reliable conclusions. Therefore, this study aimed to evaluate the clinical efficacy of VR-based pulmonary rehabilitation in COPD patients.

## 2. Materials and methods

The reporting of this meta-analysis adhered to the Preferred Reporting Items for Systematic Reviews and Meta-Analyses Statement,^[29]^ and registered in PROSPERO (CRD42023479154). Ethical approval is not necessary for meta-analysis.

### 2.1. Search strategy

We systematically retrieved databases of PubMed, EMBASE, Cochrane Library, Web of Science, and China National Knowledge Infrastructure databases from inception to March 2023. There were no restrictions on language and year of publication. The search strategy was based on MESH terms in combination with keywords using Boolean “AND” and “OR” operators: (VR OR virtual reality OR virtual environment OR videogame) AND (chronic obstructive pulmonary disease OR COPD OR pulmonary emphysema OR chronic bronchitis). The references of relevant studies were also reviewed to find eligible studies that met the inclusion and exclusion criteria. The search strategy was described in Supplemental Digital Content (see Table S1, Supplemental Digital Content, http://links.lww.com/MD/L157, which reported the search strategy for this meta-analysis).

### 2.2. Inclusion and exclusion criteria

The criteria were performed using the PICO frame: Population: patients were diagnosed with COPD (aged 18 years or older); Intervention: the intervention groups received either VR in combination with pulmonary rehabilitation therapy; Comparison: the control groups received pulmonary rehabilitation therapy alone; Outcome: forced expiratory volume in 1 second (FEV1), FEV1% predicted, FEV1/forced vital capacity (FEV1/FVC), 6-minute walking test, depression, and anxiety; Design: randomized control trials (including cluster-randomized control trials). The exclusion criteria included the following: Duplicated publications; Reviews, case reports, conference abstracts, or letters.

### 2.3. Data extraction and quality assessment

Two authors independently extracted information from selected studies using a standardized form. Any disagreements were resolved through discussion with a third author until a consensus was reached. The extracted information included: the first author, publication year, country of origin, patient demographics, the details of the intervention and control group, and the outcome index. Risk of bias was determined using the Cochrane risk of bias tool for randomized controlled trials, which included random sequence generation, allocation concealment, blinding, incomplete outcome data, selective reporting, and other potential risk of bias.

### 2.4. Data synthesis and analysis

Quantitative synthesis was conducted using Stata 12.0 (Stata Corporation, College Station, TX). Continuous data were assessed by calculating standardized mean differences (SMD) with 95% confidence intervals (CI). The threshold for statistical significance was 0.05. Heterogeneity across trials was assessed using the Cochran Q test and *I*^2^ statistics: 25%, 50%, and 75% of *I*^2^ corresponds to low, moderate, and high levels of heterogeneity respectively.^[30]^ Der Simonian-Laird random-effects model was applied when *I*^2^ > 50%. Otherwise, Mantel-Haenszel fixed-effects model was used when *I*^2^ < 50%. Begg and Egger regression test was used to assess publication bias, with *P* values of < .05 indicating potential publication bias. The sensitivity analyses were performed to evaluate the stability of the pooled results.

## 3. Results

### 3.1. Literature search

**Figure 1.**
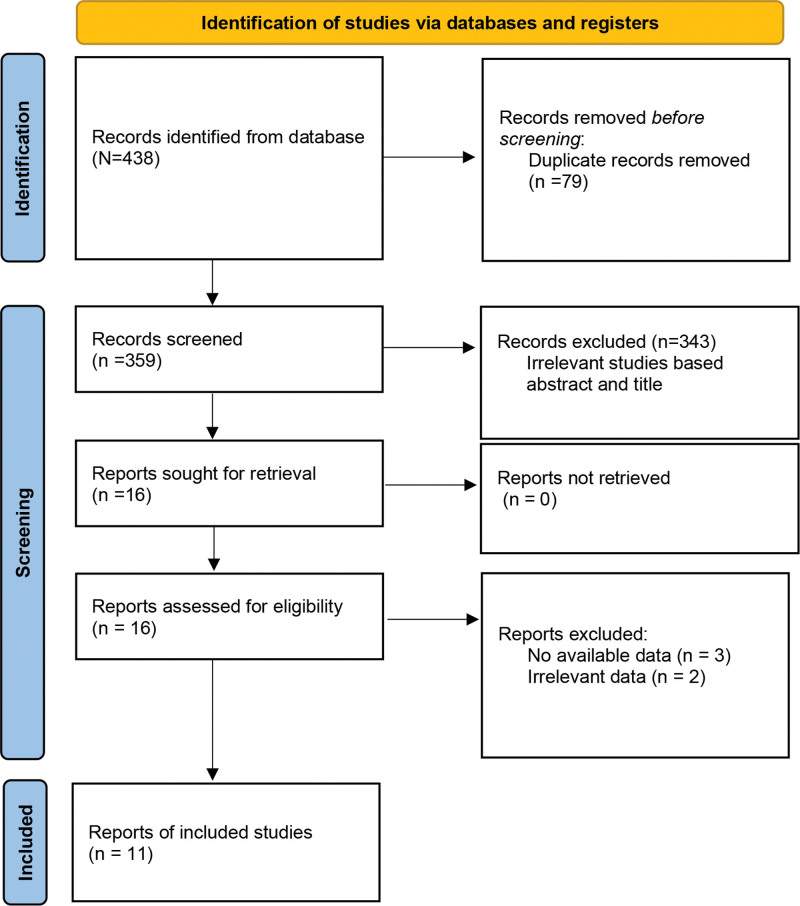
Flowchart of the article selection process.

The initial search identified 438 articles through electronic and manual database searches. After removing duplicates, 359 validated papers were identified. Reviews, case reports, conference abstracts, or irrelevant studies were excluded by reading title and abstract. The remaining 16 articles were further evaluated by full-text reading. Moreover, 5 articles were excluded due to did not meet the inclusion criteria. Finally, a total of 11 studies^[26,27,31–39]^ were included in the meta-analysis (Fig. 1).

### 3.2. Study characteristics and methodological quality

**Figure 2.**
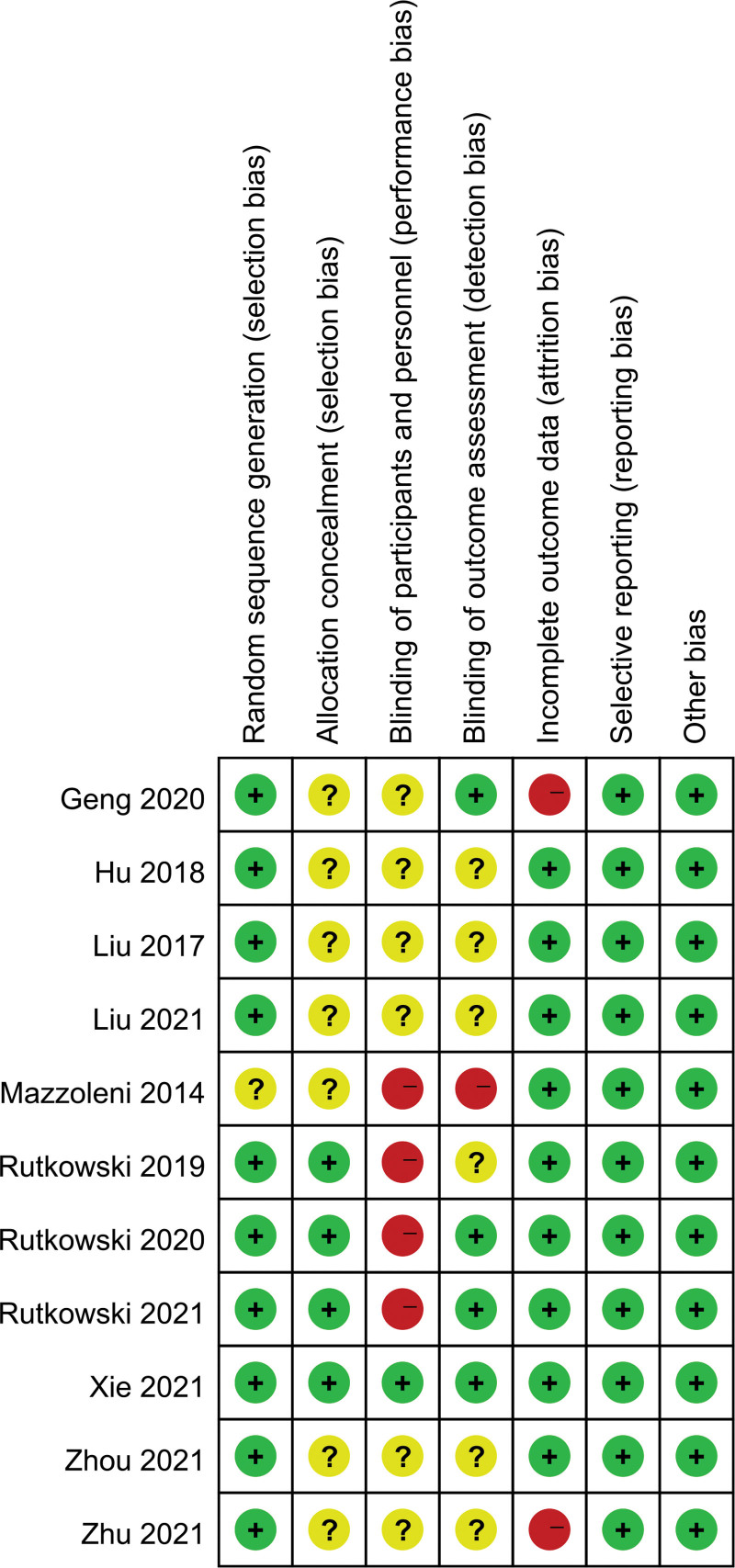
Summary of risk of bias for each included study.

The 11 studies (consisting of 751 COPD patients) included in this meta-analysis had an randomized controlled trials (RCT) design. The publication year was between 2014 and 2021. The median age of included patients ranged in age from 60.5 to 74.60 years. The sample sizes ranged from 20 to 119 across the studies. Seven studies were conducted in China, 3 in Poland, and 1 in Italy. VR-based rehabilitation is mainly divided into BioMaster virtual situational interactive training systems, Wii, Xbox Kinect, and VR TierOne. Details of the clinical characteristics of the participants and the interventions are shown in Table 1. The risk of bias in the included studies is shown in Figure 2. Random sequence generation was assessed as low risk in 90.0% (10/11) of the trials. Allocation concealment was assessed as low risk in 36.4% (4/11) of the trials. Blinding of the participants was reported in 9% (1/11) of the trials, and blinding of the outcome assessors was reported in 36.4% (4/11) of the trials. Incomplete outcome data were assessed as low risk in 81.8% (9/11) of the trials. The risk of bias for all included studies (100%) was low in selective reporting outcomes (Fig. 2).

**Table 1:** Basic characteristics of the included studies.

Author, yr	Country	Gender (F/M)	Age (yr)	Sample size	Intervention	Outcome
Liu, 2021	Chia	Exp:10/40 Con:38/12	Exp: 73.6 Con: 74.6	Exp: 50 Con: 50	Exp: received lung rehabilitation training using the BioMaster virtual scene interactive rehabilitation training system, 5–15 min/d for 12 wk Con: received routine lung rehabilitation training	FEVl, 6MWT, CAT
Rutkowski, 2020	Poland	Exp:28/10 Con:16/18	Exp: 60.6 ± 4.3 Con: 62.1 ± 2.9	Exp: 38 Con: 34	Exp: received endurance exercise training and virtual reality training using the Kinect system training, 15–30 min, five times a wk for 2 wk Con: received traditional pulmonary rehabilitation program	6MWT
Rutkowski, 2019	Poland	Exp:17/17 Con:16/18	Exp: 60.5 ± 4.3 Con: 62.1 ± 2.9	Exp: 34 Con: 34	Exp: received traditional pulmonary rehabilitation program and virtual reality training using the Kinect training system once a d for 14 d Con: received traditional pulmonary rehabilitation program	6MWT
Rutkowski, 2021	Poland	Exp:5/20 Con:4/21	Exp: 60.5 ± 4.3 Con: 67.6 ± 9.4	Exp: 25 Con: 25	Exp: received traditional pulmonary rehabilitation program and virtual reality training using VR TierOne device, 15–30 min, five times a wk for 2 wk Con: received traditional pulmonary rehabilitation program	6MWT, FEV1, HADS-D, HADS-A
Xie, 2021	China	54/6	N/A	Exp: 30 Con: 30	Exp: received traditional lung rehabilitation combined with virtual reality technology, 20 min/d for 8 wk Con: received conventional rehabilitation	FEVl, FEVl/ FVC
Mazzoleni, 2014	Italy	N/A	Exp: 68.9 ± 11.0 Con:73.5 ± 9.2	Exp: 20 Con: 20	Exp: received traditional pulmonary rehabilitation combined with virtual reality technology using Wii Fit, 60 min/d for 7 wk Con: received Pulmonary rehabilitation program	6MWT, STAI, BDEI, mMRC
Zhu, 2021	China	Exp:7/15 Con:7/14	Exp: 65.16 ± 12.78 Con: 64.54 ± 12.89	Exp: 22 Con: 21	Exp: received traditional pulmonary rehabilitation program and virtual reality training using the Kinect system training, 15–30 min, five times a wk for 16 wk Con: received traditional pulmonary rehabilitation program	6MWT, FEV1, FEVl/ FVC, mMRC, CAT
Geng, 2020	China	Exp:17/22 Con:16/18	Exp: 62.7 ± 8.6 Con: 63.2 ± 10.4	Exp: 39 Con: 34	Exp: received traditional pulmonary rehabilitation program and virtual reality training using somatosensory interactive game training, 15–30 min, five times a wk for 8 wk Con: received traditional pulmonary rehabilitation program	HADS-D, HADS-A
Liu, 2017	China	Exp:2/19 Con:2/17	Exp: 68.66 ± 6.41 Con: 68.52 ± 5.76	Exp: 33 Con: 33	Exp: received traditional pulmonary rehabilitation program and virtual reality training using s BioMaster virtual scene training system, 5–20 min, five times a wk for 8 wk Con: received traditional pulmonary rehabilitation program	6MWT, FEV1, FEVl/ FVC, CAT
Hu, 2018	China	Exp:6/14 Con:7/23	Exp: 73.6 ± 6.3 Con: 74.6 ± 5.3	Exp: 30 Con: 30	Exp: received traditional pulmonary rehabilitation program and virtual reality training using s BioMaster virtual scene training system, 5–15 min, five times a wk for 12 wk Con: received traditional pulmonary rehabilitation program	6MWT, FEV1, FEVl/ FVC, CAT
Zhou, 2021	China	Exp:13/48 Con:12/46	Exp: 70.54 ± 6.89 Con: 71.38 ± 5.78	Exp: 61 Con: 58	Exp: received traditional pulmonary rehabilitation program and virtual reality training using the Kinect system training, 30 min, five times a wk for 24 wk Con: received traditional pulmonary rehabilitation program	6MWT, FEV1, FEVl/ FVC, mMRC

6MWD = 6-minute walking distance, BEDI = beck depression inventory, CAT = COPD assessment test, FEV1 = forced expiratory volume in 1 second, FEVl/ FVC = forced expiratory volume in 1 second/forced vital capacity, HADS-D = Hospital Anxiety and Depression Scale - depression subscale, HADS-A = Hospital Anxiety and Depression Scale - anxiety subscale, mMRC = modified Medical Research Council dyspnea scale, STAI = State and Trait Anxiety Inventory, VR = virtual reality.

### 3.3. Meta-analysisa

#### 3.3.1. FEV1% predicted.

**Figure 3.**
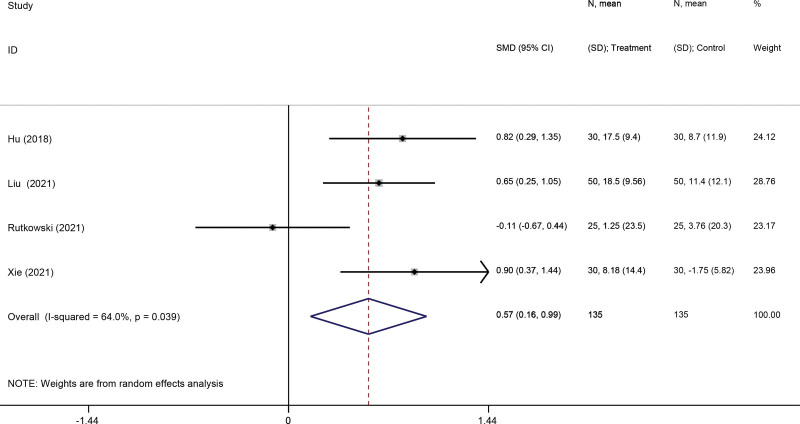
Forest plot for the effect of virtual reality-based pulmonary rehabilitation on FEV1% predicted in COPD patients. FEV1 = forced expiratory volume in 1 second, COPD = chronic obstructive pulmonary disease.

A total of 4 studies^[27,32,33,37]^ reported FEV1 as an outcome. Due to the obvious heterogeneity (*I*^2^ = 64%, *P* = .0039), a random-effect model was adopted. Pooled analysis of the 7 studies showed that VR-based pulmonary rehabilitation had a significant effect in FEV1% predicted compared to pulmonary rehabilitation alone (SMD: 0.57; 95% CI 0.16,0.99; *P* = .007) (Fig. 3).

#### 3.3.2. FEV1.

**Figure 4.**
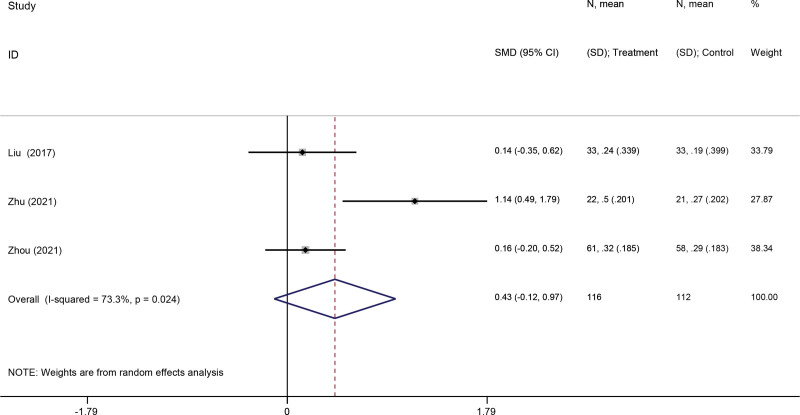
Forest plot for the effect of virtual reality-based pulmonary rehabilitation on FEV1 in COPD patients. FEV1 = forced expiratory volume in 1 second, COPD = chronic obstructive pulmonary disease.

Three studies^[34,38,39]^ reported FEV1% predicted as an outcome. Due to the obvious heterogeneity (*I*^2^ = 73.3%, *P* = .024), a random-effect model was used. Meta-analysis revealed that VR-based pulmonary rehabilitation was less effective in improving the FEV1 of COPD patients than the control group (SMD: 0.43; 95% CI −0.12,0.97; *P* = .124) (Fig. 4).

#### 3.3.3. FEV1/FVC.

**Figure 5.**
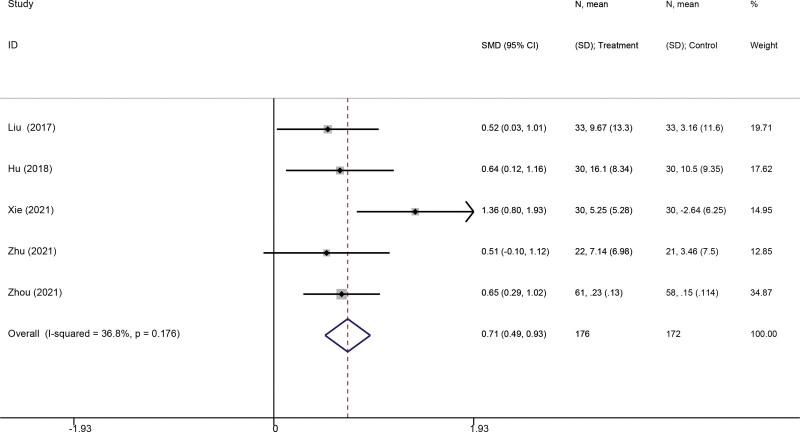
Forest plot for the effect of virtual reality-based pulmonary rehabilitation on FEV1/FVC in COPD patients. FEV1/FVC = forced expiratory volume in 1 second/forced vital capacity. COPD = chronic obstructive pulmonary disease.

Four studies^[27,32,34,39]^ were eligible for FEV1/FVC analysis. Pooled analysis showed that VR-based pulmonary rehabilitation made statistically significant improvements in FEV1/FVC (SMD: 0.71; 95% CI 0.49, 0.93; *P* < .001), compared to traditional rehabilitation (Fig. 5).

#### 3.3.4. Dyspnoea.

**Figure 6.**
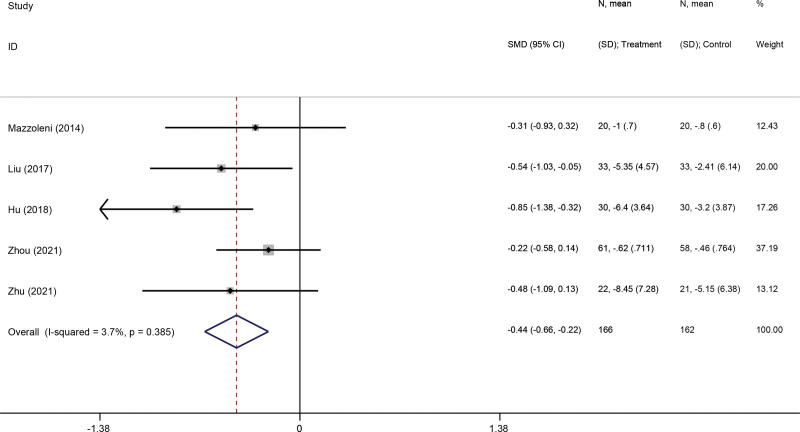
Forest plot for the effect of virtual reality-based pulmonary rehabilitation on dyspnea in COPD patients. COPD = chronic obstructive pulmonary disease.

MRC and CAT were used to evaluate the dyspnea of the COPD patients. Two studies^[35,38]^ reported the MRC and 3 studies^[32,34,39]^ reported the CAT. Compared with pulmonary rehabilitation alone, the use of VR-based pulmonary rehabilitation was associated with significant improvements in dyspnea (SMD: −0.44; 95% CI −0.66, −0.22; *P* < .001) (Fig. 6).

#### 3.3.5. 6MWD.

**Figure 7.**
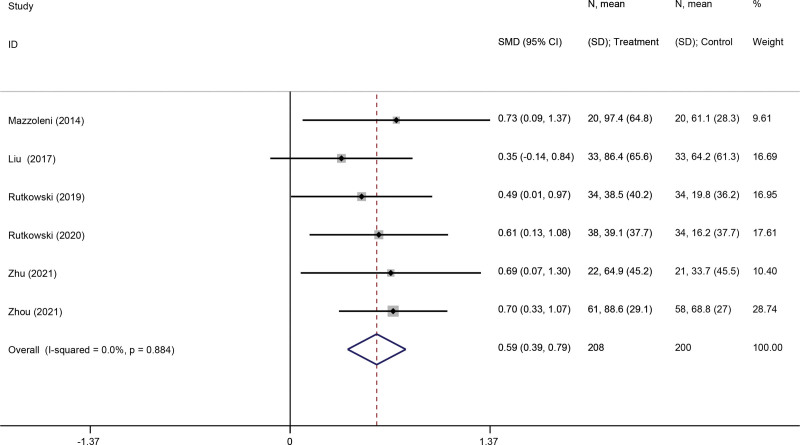
Forest plot for the effect of virtual reality-based pulmonary rehabilitation on 6MWD in COPD patients. 6MWD = 6-minute walking distance, COPD = chronic obstructive pulmonary disease.

Six studies^[26,34–36,38,39]^ evaluated the impact of VR-based pulmonary rehabilitation on 6MWD of patients with COPD. Compared with the pulmonary rehabilitation alone group, the results showed that VR-based pulmonary rehabilitation had a significant effect (SMD: 059; 95% CI 0.39, 0.79; *P* < .001) (Fig. 7).

#### 3.3.6. Mood.

**Figure 8.**
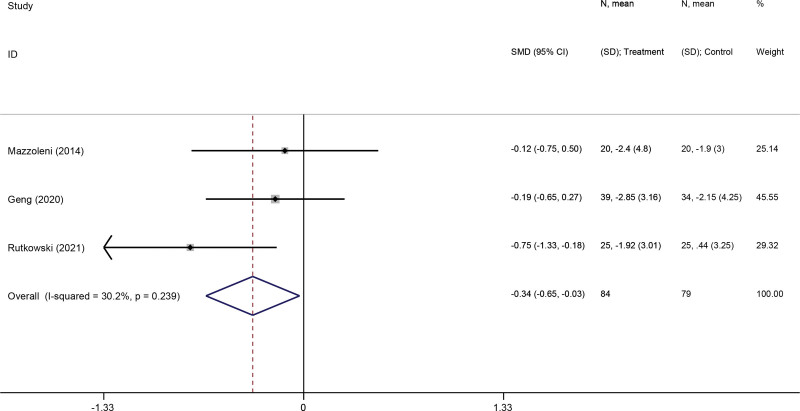
Forest plot for the effect of virtual reality-based pulmonary rehabilitation on depression in COPD patients. 6MWD = 6-minute walking distance, COPD = chronic obstructive pulmonary disease.

**Figure 9.**
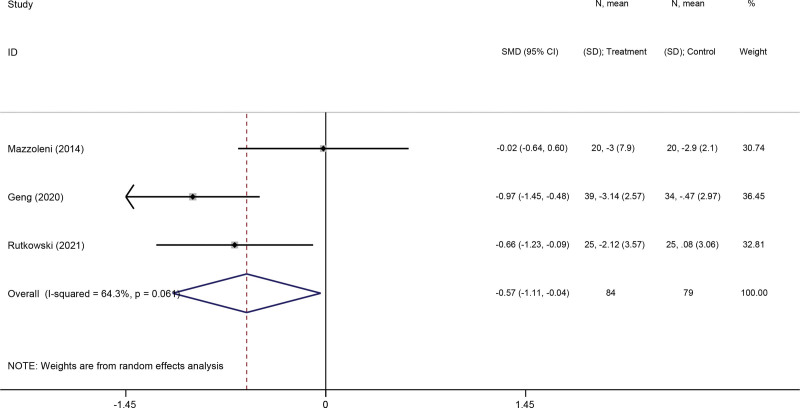
Forest plot for the effect of virtual reality-based pulmonary rehabilitation on anxiety in COPD patients. 6MWD = 6-minute walking distance, COPD = chronic obstructive pulmonary disease.

Three studies^[31,35,37]^ reported the effect of VR-based pulmonary rehabilitation on depression and anxiety of COPD patients. The result from pooled data showed that VR-based pulmonary rehabilitation was effective in improving depression (SMD: −0.34; 95% CI −0.05, −0.03; *P* = .033) (Fig. 8) and anxiety mood in COPD patients (SMD: −0.57; 95% CI −1.11, −0.04; *P* = .036) (Fig. 9).

### 3.4. Publication bias

**Figure 10.**
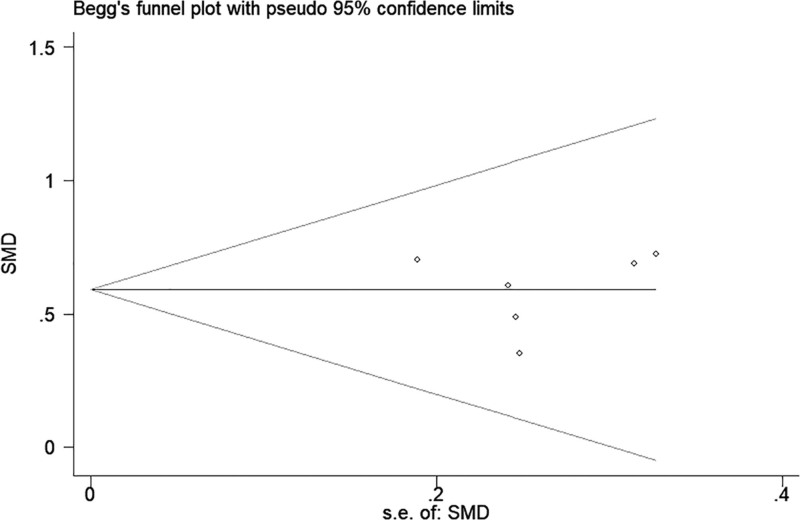
Begg funnel plot of risk of falls.

Visualized funnel plots, Begg test, and Egger tests were used to evaluate publication bias. There was no statistical evidence of the existence of publication bias in 6MWD, according to the results of these tests (Begg *P* = .851; Egger *P* = .948) (Fig. 10).

## 4. Discussion

This meta-analysis evaluates the effectiveness of VR-based pulmonary rehabilitation in the treatment of COPD patients compared to traditional rehabilitation. Despite the increasing popularity of VR in rehabilitation, its clinical practice for COPD patients is still limited. Therefore, our study aims to promote evidence-based practice. Our meta-analysis found that VR-based pulmonary rehabilitation is superior to pulmonary rehabilitation alone in improving COPD patients’ pulmonary function and exercise capacity; VR-based pulmonary rehabilitation is better than pulmonary rehabilitation alone for improving anxiety and depression in COPD patients.

The interaction with these virtual environments is different depending on the level of immersion of these devices, thus, these devices divide into non-immersive and immersive VR devices.^[40]^ Four types of devices are included in this study, including BioMaster virtual situational interactive training system, Wii, Xbox Kinect, and VR TierOne. BioMaster virtual situational interactive training system uses computer hardware and software to create interactive simulations that provide users with the opportunity to engage in environments that look and feel similar to the real world.^[33]^ Wii uses a remote control to perform actions and interact with virtual objects displayed on the screen, with or without the use of a platform called the Wii Balance Board.^[41]^ Xbox Kinect is a motion capture device that provides biofeedback as it can detect the patient’s outline.^[42]^ VR TierOne produces strong visual, auditory, and kinesthetic stimuli through a head-mounted display and complete immersion in the phenomenon.^[37]^

COPD is a chronic disease characterized by airway and alveolar abnormalities, and airflow limitation. FEV1/FVC, FEV1%, and FVC are important indicators for evaluating the lung function of COPD patients.^[43]^ We found that compared with conventional rehabilitation, VR-based pulmonary rehabilitation significantly improved FEV1% and FEV1/FVC in COPD patients. Conventional pulmonary rehabilitation, as a non-pharmacological intervention, can improve ventilation disorders and hypoxia in COPD patients, but the frequency of maintenance rehabilitation training may decrease over time.^[44]^ Personalized rehabilitation training based on VR technology allows patients to benefit from greater autonomy and activity during daily activities, thus better slowing down the progression of the disease.^[45]^

The feeling of respiratory distress is mainly caused by impaired gas exchange function in the alveoli. Three studies used the MRC scale to evaluate respiratory distress at the end of the intervention course, and 3 studies used the CAT scale. The results showed that VR-based rehabilitation was significantly better than the control group in improving respiratory distress. One study used the Borg scale to measure dyspnea, the improvement in respiratory distress was not significant. MRC and CAT belong to clinical type scales, while Borg belongs to psychological scales.^[46]^ When using the Borg scale, the patient’s emotional tendencies should be evaluated beforehand. Although the pulmonary rehabilitation guidelines recommend the use of the Borg respiratory distress scale to evaluate exercise intensity, it depends on individual understanding.^[47]^

In patients with COPD, a common occurrence is a decline in motor capacity. The 6MWT is the gold standard for assessing functional capacity in COPD patients.^[48,49]^ Our meta-analysis shows that VR-based rehabilitation can increase the average 6MWT distance, significantly improving exercise capacity. Although the minimum clinically important difference in 6MWT after VR-based pulmonary rehabilitation has not yet been determined, it is superior to the improvement seen after traditional rehabilitation. Therefore, VR-based pulmonary rehabilitation has a positive effect on 6MWT in COPD. Depression, anxiety, and other negative emotions are common in patients with COPD.^[50]^ Depression and anxiety symptoms in COPD lead to worse health outcomes, including reduced health-related quality of life and increased risk of death.^[50]^ Patients with anxiety and depression symptoms lack confidence in the success of rehabilitation and are unwilling to commit to improving their health.^[8,51]^ The effectiveness of VR therapy in reducing anxiety and depression has been demonstrated in other rehabilitation fields.^[52]^ Our research results are consistent with this, and suggest that VR-based pulmonary rehabilitation can significantly improve anxiety and depression symptoms in COPD compared to the control group.

However, this review has several limitations. Firstly, due to the small number of participants, the impact of COPD severity on the effectiveness of VR-based pulmonary rehabilitation cannot be concluded. Secondly, the included studies used semi-immersive VR therapy systems or immersive VR therapy systems, and there is insufficient data to compare the differences. Thirdly, the limited number of RCTs and sample sizes may affect the effect of VR-based therapy. Fourthly, there are no studies evaluating the long-term effects of VR-based pulmonary rehabilitation.

## 5. Conclusion

Our meta-analyses suggest that has been found to be effective in enhancing the therapeutic efficacy of pulmonary rehabilitation in patients with COPD, having a beneficial effect on lung function and mood.

However, this evaluation is limited by the availability of high-quality evidence and the variability of rehabilitation programs. More well-designed RCTs are needed to estimate the applicability and feasibility of VR-based pulmonary rehabilitation in patients with COPD, and evaluate the optimal VR-based rehabilitation protocol.

## Author contributions

**Conceptualization:** Zhihong He.

**Data curation:** Xiuqin Chai, Lingyun Wu.

**Formal analysis:** Xiuqin Chai, Lingyun Wu.

**Methodology:** Xiuqin Chai, Lingyun Wu, Zhihong He.

**Supervision:** Zhihong He.

**Writing – original draft:** Xiuqin Chai, Lingyun Wu.

**Writing – review & editing:** Xiuqin Chai, Lingyun Wu.

## Supplementary Material


