# Supplementary material for: Effects of virtual reality-based pulmonary rehabilitation in patients with chronic obstructive pulmonary disease: A meta-analysis
Source: Medicine (Baltimore). 2023 Dec 29;102(52):e36702. doi: 10.1097/MD.0000000000036702 (PMC10754576; doi:10.1097/MD.0000000000036702)
Supplement: Supplementary file 1 [file medi-102-e36702-s001.docx]

**Table S1：The details of the search strategies.**

|  | |
| --- | --- |
| Database | Search strategy |
| PUBMED | ("vis resour"[Journal] OR "proc ieee virtual real conf"[Journal] OR "vr"[All Fields] OR ("virtual reality"[MeSH Terms] OR ("virtual"[All Fields] AND "reality"[All Fields]) OR "virtual reality"[All Fields]) OR (("virtual"[All Fields] OR "virtuality"[All Fields] OR "virtualization"[All Fields] OR "virtualized"[All Fields] OR "virtualizing"[All Fields] OR "virtuals"[All Fields]) AND ("environ"[All Fields] OR "environment"[MeSH Terms] OR "environment"[All Fields] OR "environments"[All Fields] OR "environment s"[All Fields] OR "environs"[All Fields])) OR ("videogame"[All Fields] OR "videogames"[All Fields] OR "videogaming"[All Fields])) AND ("pulmonary disease, chronic obstructive"[MeSH Terms] OR ("pulmonary"[All Fields] AND "disease"[All Fields] AND "chronic"[All Fields] AND "obstructive"[All Fields]) OR "chronic obstructive pulmonary disease"[All Fields] OR ("chronic"[All Fields] AND "obstructive"[All Fields] AND "pulmonary"[All Fields] AND "disease"[All Fields]) OR ("pulmonary disease, chronic obstructive"[MeSH Terms] OR ("pulmonary"[All Fields] AND "disease"[All Fields] AND "chronic"[All Fields] AND "obstructive"[All Fields]) OR "chronic obstructive pulmonary disease"[All Fields] OR "copd"[All Fields]) OR ("pulmonary emphysema"[MeSH Terms] OR ("pulmonary"[All Fields] AND "emphysema"[All Fields]) OR "pulmonary emphysema"[All Fields]) OR ("bronchitis, chronic"[MeSH Terms] OR ("bronchitis"[All Fields] AND "chronic"[All Fields]) OR "chronic bronchitis"[All Fields] OR ("chronic"[All Fields] AND "bronchitis"[All Fields])))  Identified 93 articles |
| The Cochrane Library | ((VR):ti,ab,kw OR (virtual reality):ti,ab,kw OR (virtual environment):ti,ab,kw OR (videogame):ti,ab,kw OR MeSH descriptor: [virtual reality] in all MeSH products) AND ((chronic obstructive pulmonary disease):ti,ab,kw OR (COPD):ti,ab,kw) AND ((pulmonary emphysema):ti,ab,kw OR (chronic bronchitis):ti,ab,kw OR MeSH descriptor: [chronic obstructive pulmonary disease] explode all trees)  Identified 37 articles |
| EMBASE | (' virtual reality '/exp OR VR.ti,kw,hw OR virtual reality.ti,kw,hw OR 'VR'/exp OR' virtual environment '/exp OR virtual environment.ti,kw,hw' OR ' videogame '/exp OR videogame.ti,kw,hw)  AND (' chronic obstructive pulmonary disease '/exp OR chronic obstructive pulmonary disease.ti,kw,hw OR ' COPD '/exp OR COPD. ti,kw,hw OR ' pulmonary emphysema '/exp OR pulmonary emphysema.ti,kw,hw' OR ' chronic bronchitis '/exp OR chronic bronchitis. ti,kw,hw)  Identified 238 articles |
| Web of science | ((((TS=(VR)) OR TS=( virtual reality))) OR TS=(virtual environment )) OR TS=( videogame) AND (((TS=(chronic obstructive pulmonary disease )) OR TS=(COPD)) OR TS=(pulmonary emphysema)) OR TS=(chronic bronchitis) |
|  | Identified 56 articles |
| CNKI | (SU=“VR” OR SU=“virtual reality” OR SU=“virtual environment” OR SU=“videogame”) AND (SU=“COPD” OR SU=“chronic obstructive pulmonary disease” OR SU=“chronic bronchitis” OR SU=“pulmonary emphysema”)  Identified 14 articles |
